# Intravenous rehydration in children with severe malnutrition and severe dehydration: a systematic review and meta-analysis

**DOI:** 10.1136/archdischild-2026-330358

**Published:** 2026-09-18

**Authors:** Juan Emmanuel Dewez, San Maurice Ouattara, Temmy Sunyoto, Christabel Kemunto Mogaka, Matthew E. Coldiron, Hadiza Alhaji Sainna, Roberta Petrucci, Elizabeth C George, Kathryn Maitland

**Affiliations:** 1 London School of Hygiene and Tropical Medicine, London, UK; 2 Epicentre Niger, Magaria, Niger; 3 Medecins Sans Frontieres, Brussels, Belgium; 4 KEMRI-Wellcome Trust Research Programme, Kilifi, Kenya; 5 Epicentre, Paris, France; 6 Medecins Sans Frontieres, Maiduguri, Nigeria; 7 Paediatric Emergency Unit, Department of Paediatrics, Medecins Sans Frontieres, Geneva, Switzerland; 8 Medical Research Council Clinical Trials Unit at University College London, London, UK; 9 Institute of Global Health Innovation, Department of Surgery and Cancer, Imperial College London, London, UK

## Abstract

**Objectives:**

To compare the efficacy and safety of intravenous rehydration (IVR) versus oral rehydration (OR) strategies in children with severe acute malnutrition (SAM) and severe dehydration.

**Design:**

Systematic review and meta-analysis.

**Setting:**

Lower-to-middle-income countries.

**Patients:**

Children with SAM are hospitalised with severe dehydration secondary to gastroenteritis.

**Interventions:**

Randomised trials of IVR and OR (standard of care)

**Main outcome measures:**

Primary: in-hospital mortality. Secondary: fluid overload events, development of shock requiring intravenous boluses, severe electrolyte abnormalities at 24 hours and day 28 mortality.

**Results:**

We identified three RCTs comprising 484 participants with severe malnutrition (72 had kwashiorkor, two had some risk of bias, and one had low risk of bias). The pooled risk ratio (RR) for in-hospital mortality for IVR versus OR is 0.71 (95% CI 0.46 to 1.10; I^2^=0.0%) with moderate certainty of evidence. No fluid overload events were reported, pooled RR 0.99 (95% CI 0.10 to 9.35). The pooled RR of severe hyponatraemia (sodium <125 or <130 mmol/L) at 24 hours was 0.66 (95% CI 0.44 to 0.99). Only one trial reported RR for shock development, hypernatraemia (sodium >145 mmol/L) or 28-day mortality with IVR versus OR (RRs 0.56, 95% CI 0.21 to 1.48; RR 2.05, 95% CI 0.50 to 8.58 and RR 0.85, 95% CI 0.44 to 1.65, respectively). Subgroup analyses for in-hospital mortality were carried out for region and risk of bias rating, giving p=0.85 and p=0.54 for heterogeneity, respectively.

**Conclusions:**

The estimated effect of using IVR versus OR in children with SAM with severe dehydration ranges from a 54% relative reduction to a 10% relative increase in the risk of death, with IVR resulting in fewer adverse events.

**PROSPERO registration number:**

CRD42025637956.

## Introduction

Current international recommendations for rehydration for the inpatient management of children with severe dehydration and severe acute malnutrition (SAM) differ from those without SAM.^1^ These advise against the use of intravenous rehydration (IVR). Thus, oral rehydration (OR) is the recommended option, with intravenous boluses provided only in the event of shock, resulting in children requiring a nasogastric tube to be administered OR, since most are unable to take or retain oral fluids. As most cases are managed on busy, overcrowded paediatric wards with limited numbers of clinical staff, safe implementation of oral-nasogastric rehydration and monitoring for signs of shock remain challenging. The recommendations are based on concerns about children with SAM being at high risk of cardiac compromise and sodium overload.^2 3^ While physiological studies have supported the safety of intravenous fluids in SAM and found no evidence of cardiac compromise,^4 5^ including appropriate adaptive responses,^4 6^ controlled clinical trials assessing the safety and efficacy of liberal intravenous rehydration are lacking.^7^

Severe acute malnutrition remains a leading cause of paediatric hospital admissions in Africa,^8 9^ and dehydration due to gastroenteritis is a common complication in children with SAM and is associated with poor outcomes reported,^8 10 11^ suggesting that the current standard management might be suboptimal. The recent results from Gastroenteritis: Rehydration of children with Severe Acute Malnutrition (GASTROSAM),^12^ the largest trial to date conducted in Africa addressing this question, justify a comprehensive re-examination of the evidence.

Our objective was to systematically review the literature and assess the efficacy and safety of intravenous rehydration compared with standard care (OR) in children hospitalised with SAM complicated by severe dehydration due to gastroenteritis.

## Methods

This systematic review and meta-analysis was conducted following the Preferred Reporting Items for Systematic Reviews and Meta-Analyses (PRISMA) recommendations. The review protocol was submitted to the PROSPERO repository (CRD42025637956) before the review was conducted.

### Eligibility criteria

We included randomised controlled trials (RCTs) and quasi-randomised controlled trials evaluating children aged 5 months to 12 years hospitalised with severe malnutrition (defined as any one of the following: weight-for-height Z-score less than −3 or mid-upper arm circumference (MUAC) less than 11.5 cm or oedematous malnutrition (kwashiorkor: bilateral pedal oedema or more generalised oedema) with gastroenteritis/diarrhoea (>3 loose stools/day) and severe dehydration (following WHO criteria, including two or more of the following: altered consciousness (less than alert on AVPU score), sunken eyes, reduced skin turgor (slow abdominal skin pinch return >2 s) or inability to take or retain oral fluids). The trials compared, by intention, one group rehydrated intravenously with fluids of any type and volume (intervention group; IVR) to an OR strategy (defined as the control). As per WHO guidelines, the control group were permitted to receive intravenous fluids to treat hypovolaemic shock (defined as any of capillary refill >3 s, weak pulse or cool peripheries); OR was permitted in all children (as a follow-on from IVR for children with ongoing losses). We excluded studies which enrolled children with known uncorrected congenital heart abnormalities or pre-existing renal conditions.

### Outcomes

The prespecified primary outcome was in-hospital mortality. Secondary outcomes were fluid overload events (pulmonary oedema or heart failure), development of shock requiring intravenous boluses, development of neurological complications (convulsions or decrease in conscious level), presence of severe electrolyte abnormalities (hyponatraemia, hypernatraemia or hypokalaemia as defined by the study), day-28 mortality and hospital readmission.

### Information sources

MEDLINE, Embase, CINAHL and Cochrane Central Register of Controlled Trials (CENTRAL, the Cochrane Library) were searched. We searched for relevant references cited in the papers identified through the above databases. We also searched for ongoing clinical trials through ClinicalTrials.gov, the ISRCTN registry and the WHO International Clinical Trials Registry Platform to include potentially validated data in the process of being published but not yet accessible. The last searches were conducted on 23 November 2025.

### Search strategy and study selection

The search strategy was based on intravenous fluid management, children, severe acute malnutrition, dehydration and diarrhoea (or gastroenteritis). We listed key search terms, their synonyms and Medical Subject Headings (MeSH) for each concept. We then conducted the search combining the key search terms, their synonyms and MeSH with appropriate Boolean operators (online supplemental tables S1–S4). No time or language restrictions were applied.

Search results were imported into a reference management software (Zotero). Duplicates were removed through a specific feature of the software. Paired reviewers (MC, SMO, HAS and TS) independently conducted title and abstract screening (including full-text screening when titles and abstracts provided insufficient information). MC and SMO screened half of the articles; HAS and TS screened the other half. Discrepancies were resolved through consensus by discussion with an additional reviewer (JED). Screening was based on the eligibility criteria described above.

### Data extraction

A standardised data extraction form was used to extract data needed to describe the included articles, assess their quality and perform the meta-analysis. The data included publication characteristics, country, baseline demographics (including SAM definition and dehydration definition), study design, inclusion criteria, exclusion criteria, description of intervention, description of control care, number of participants assigned to each group and the predefined outcomes of each study and reported subgroup analyses. The outcome data included the number randomised, the number with an outcome assessed and the number with an outcome of interest to estimate frequencies and percentages.

Data were extracted by the paired reviewers. Discrepancies were resolved through consensus by discussion with an additional reviewer (JED).

### Risk-of-bias assessment

Paired reviewers (JED, CKM and TS) conducted risk-of-bias assessments independently (JED assessed all papers and CKM and TS each assessed one half of the papers independently) using the Risk of Bias 2.0 tool.^13^ Discrepancies were solved through consensus discussions between JED, CKM and TS. Risk-of-bias was classified as low, high or some concerns for the primary outcome (mortality before discharge). The assessment was based on the randomisation process, deviations from the intended intervention, missing outcome data, measurement of the outcome and selection of the reported result as per the Risk of Bias 2.0 tool instructions.

### Statistical analyses

Meta-analyses were performed in Stata V.18.0 with random effects and using restricted maximum likelihood for risk ratios (RRs) and DerSimonian–Laird for risk differences (RDs) where there were studies with zero events. All outcomes compare intravenous rehydration (experimental treatment) versus OR (control: standard of care) and are presented as RRs and RDs. Only outcomes reported in two or more studies were analysed. Heterogeneity between studies was assessed using I^2^ and visual inspection of forest plots. Prespecified subgroup analyses included for the primary outcome (in-hospital mortality) compared trials in Africa versus Asia, children under 1 year and the presence of kwashiorkor. One planned subgroup analysis for the primary outcome (in-hospital mortality) was conducted by region (comparing Africa and Asia), and a post hoc subgroup analysis by risk of bias was performed as part of the Grading of Recommendations Assessment, Development, and Evaluation (GRADE) assessment. Other prespecified subgroups based on age and presence of kwashiorkor (oedematous malnutrition) could not be analysed as the specific data were not disaggregated in the published reports.

### Certainty of evidence

The overall certainty in evidence for each outcome of interest, summarised with meta-analysis, was rated using the GRADE framework, based on risk of bias, imprecision, inconsistency, indirectness and publication bias. The certainty of evidence was rated as very low, low, moderate or high.^14^

## Results

### Study selection

**Figure 1:**
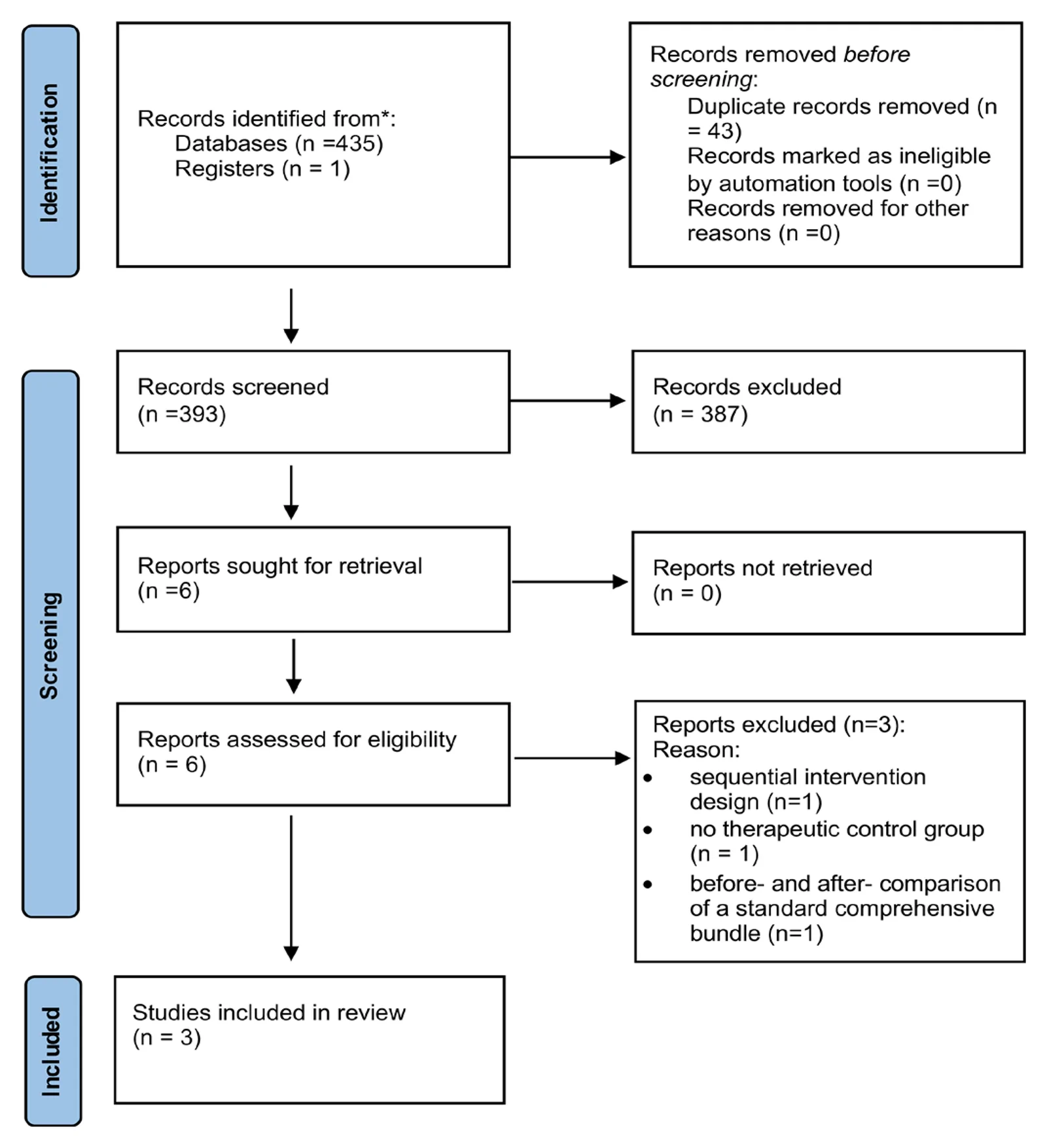
Preferred Reporting Items for Systematic Reviews and Meta-Analyses flow diagram.

Of 393 unique citations identified by the search, six studies^12 15–19^ were assessed for potential eligibility after the initial screening (figure 1). Articles not found to meet the study inclusion criteria were excluded, with the reasons recorded. Three studies were excluded, as they were not RCTs: one study was excluded because it was a non-randomised observational cohort study reporting before-and-after implementation of a therapeutic bundle^15^; a second study did not include a control group (OR),^16^ and a third study was excluded, as it was a sequential intervention study design^17^ (online supplemental table S5). Of the three studies remaining, only one trial fulfilled the strict criteria outlined in the search strategy (PROSPERO CRD42025637956). Two other trials^18 19^ examined a more liberal intravenous rehydration strategy and a more conservative strategy, so they were included in the meta-analysis for completeness. One study recruited SAM children with severe dehydration or septic shock^18^; however, data were presented separately, and only data for children with severe dehydration were used in this analysis. Another study recruited children with moderate or severe acute malnutrition^19^; however, only SAM children are included in the analysis. These three studies contributed 484 children in total (figure 1 and table 1).

### Study characteristics

The three included studies were an RCT conducted in Kenya,^18^ an RCT conducted in Bangladesh^19^ and a multinational RCT conducted in Kenya, Uganda, Niger and Nigeria^12^ (table 1 and online supplemental table S6a,b). The RCTs all enrolled children >6 months, with no reported upper limit for Akech *et al* (median 15 and 16 months in intervention and control arms, respectively), Alam *et al* to 60 months (median 30 and 32 in intervention and control, respectively) and Maitland *et al* to 12 years (median 13 months). This RCT included children with SAM and chronic malnutrition^19^; however, the other two studies included only children with SAM.^12 18^ The kwashiorkor phenotype of severe malnutrition was present in 12 (22%)^18^ of study participants in Akech *et al*, 49 (24%)^19^ in Alam *et al* and 11 (4%)^12^ in Maitland *et al*.^12^ All three trials included children with severe dehydration. In Maitland *et al*, 12/102 (12%) children were complicated by invasive bacterial infection (largely due to gram-negative bacteraemia).^12^ Akech *et al* enrolled children with severe dehydration and hypovolaemic shock (40/61 participants) and septic shock without dehydration (21/61 participants).^18^ The data from the latter group were excluded from this systematic review. Akech *et al* compared providing intravenous boluses of Ringer’s lactate (RL) (up to 40 mL/kg) (intervention) versus 1–2 intravenous boluses of 15 mL/kg intravenous half-strength Darrow’s/5% dextrose (HSD/5D) (control). Oral rehydration solution for malnutrition (ReSoMal) was given to children with significant diarrhoea in both arms.^18^ Alam *et al* compared the provision of 100 mL/kg of an isotonic intravenous solution over 6 hours (intervention) versus up to 30 mL/kg given over 2 hours (same solution) followed by an OR (control).^19^ Maitland *et al* evaluated three interventions: immediate rapid rehydration (100 mL/kg RL over 3–6 hours according to age), immediate slow rehydration (100 mL/kg RL given over 8 hours) (both considered as interventions) versus control (OR solution at a rate of 5 mL/kg every 30 min for the first 2 hours followed by 5–10 mL/kg per hour for 4–10 hours). No IVR was permitted in the control arm; per protocol, fluid boluses were reserved for those with or developing shock^12^ (online supplemental table S6b). The availability of the primary and secondary outcomes in the three trials is summarised in online supplemental table S7.

### Risk of bias in studies

**Figure 2:**
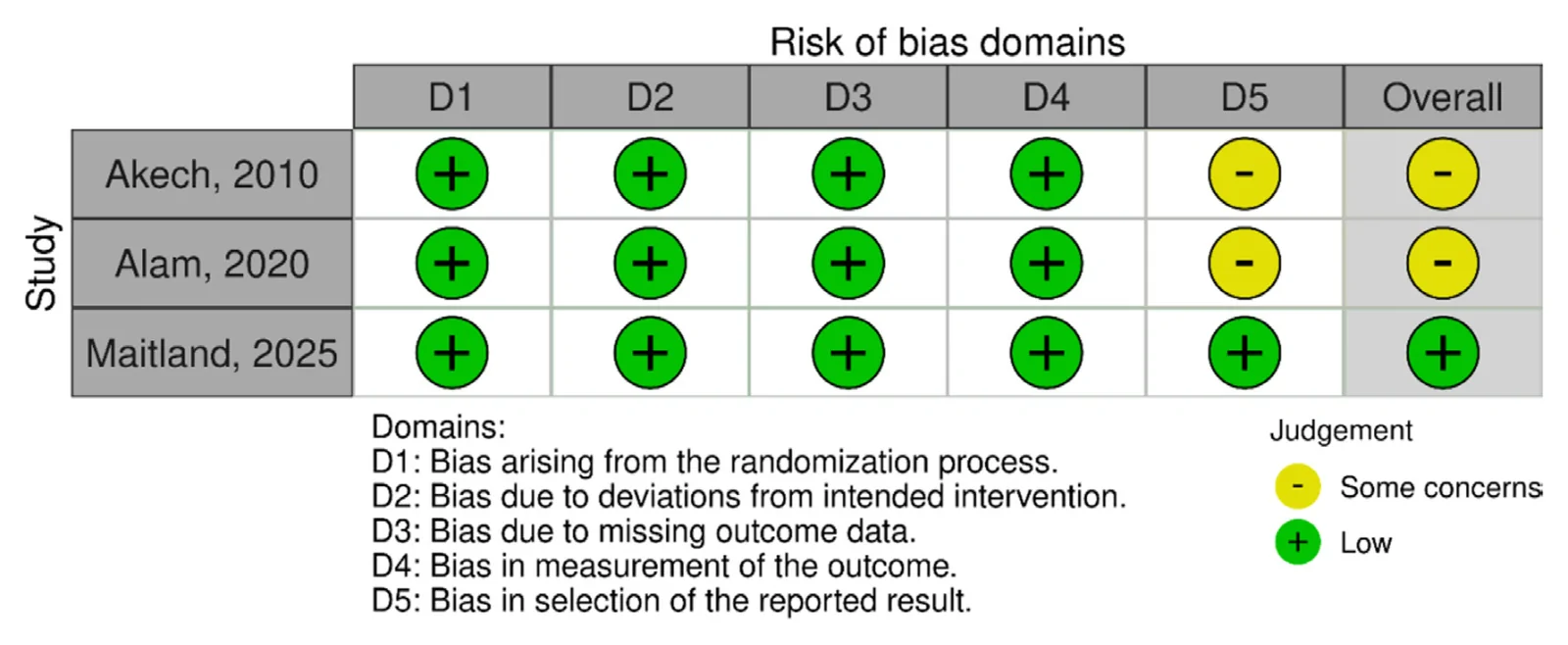
Risk of bias plot.

Overall, two studies^18 19^ were assessed as presenting some concerns on risk of bias domains, as there was risk in the selection of reported results, which may have resulted in bias. For Akech *et al*, the timing of the measurement of the primary outcome in their ISRCTN registration did not match what was reported in the publication. For Alam *et al*, the trial was retrospectively registered. Maitland *et al*’s RCT had low risk of bias^12^ (figure 2)

### Primary outcome

**Figure 3:**
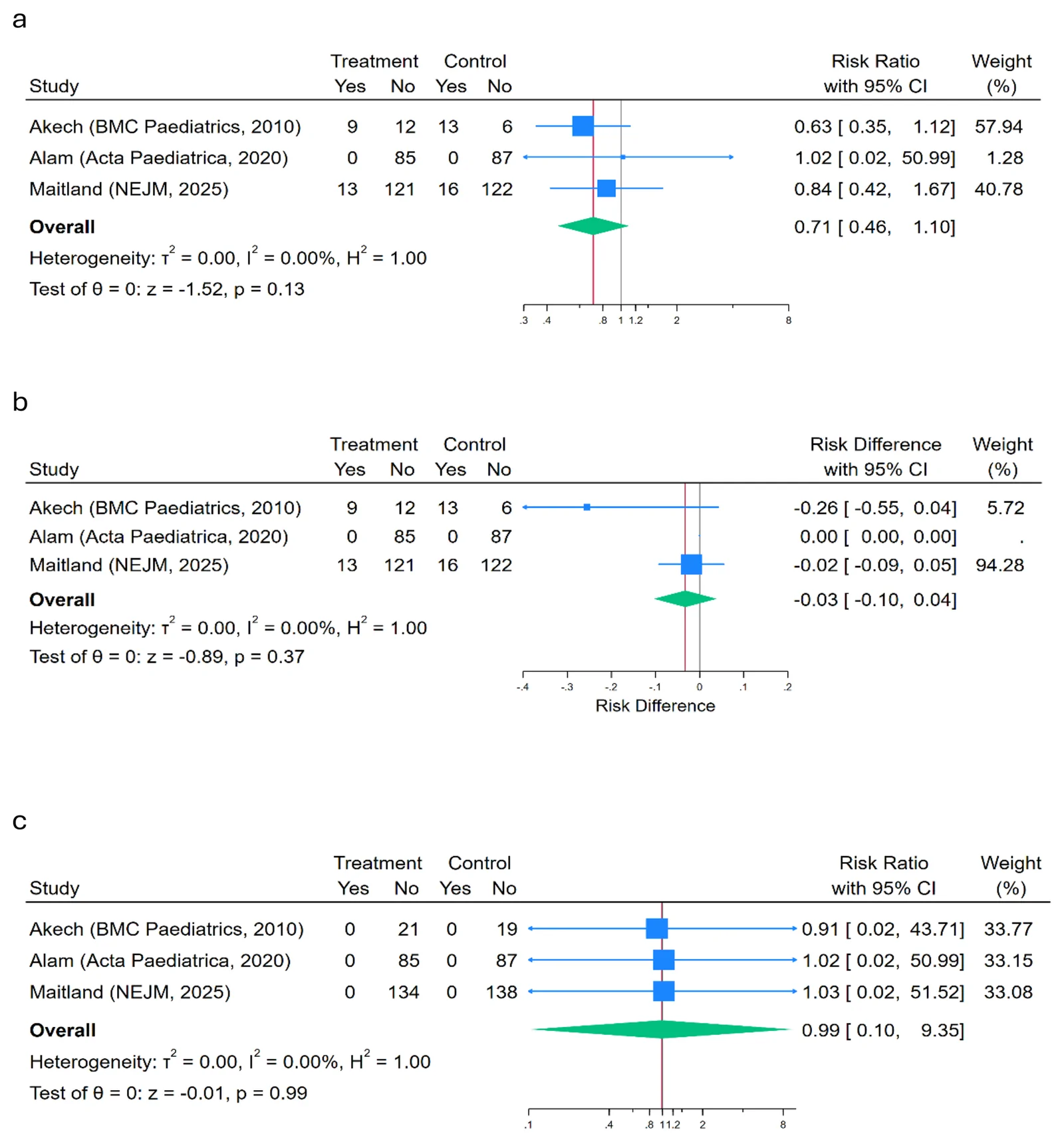
Forest plots. (a) Risk ratio and (b) risk difference for in-hospital mortality and (c) risk ratio for heart failure or fluid overload events. NB: Due to no events in any study, the forest plot for the risk difference was not estimated.

The pooled estimated RR for in-hospital mortality was 0.71 (95% CI 0.46 to 1.1) I^2^=0.0% with a pooled RD of −3.2% (95% CI −10.4 to 3.9) (figure 3a,b, table 2 and online supplemental table S8). This translates to an estimated effect for IVR versus OR ranging from a 54% relative reduction to a 10% relative increase in the risk of in-hospital mortality. The GRADE summary indicates moderate certainty of evidence for the primary outcome, with no evidence of a difference between intervention and control strategies for rehydration of children with severe acute malnutrition and severe dehydration.

### Secondary outcomes

No fluid overload events were reported in any RCT; the pooled RR for fluid overload in IVR versus OR was 0.99 (95% CI 0.10 to 9.35) (figure 3c and table 2) with moderate certainty of evidence. Severe hyponatraemia and hypokalaemia at 24 hours were reported in two studies, with different thresholds (as <125 mmol/L and <2.5 mmol/L, respectively,^12^ and <130 and <3.5, respectively.^19^ The different thresholds were defined as subgroups, and the pooled estimated RR for IVR versus OR was 0.66 (95% CI 0.44 to 0.99) for hyponatraemia (online supplemental figure S1) with moderate certainty of evidence and 1.16 (95% CI 0.93 to 1.46) with low certainty of evidence for hypokalaemia (online supplemental figure S2).

Other prespecified outcomes were only reported in one RCT,^12^ so they were not pooled for analysis, including development of shock, development of neurological complications (convulsions or decrease in conscious level) during the primary admission, day 28 mortality and severe hypernatraemia (>145 mmol/L). In Maitland *et al*, for the pooled IVR versus OR comparison for shock development, occurring in 6/121 (5%) vs 11/126 (9%), respectively, the RR was 0.56 (95% CI 0.55 to 1.53); hypernatraemia (sodium >145 mmol/L) occurred in 6/127 versus 3/128, respectively, with RR 2.05 (95% CI 0.50 to 8.58). Day 28 mortality was reported in 14/134 versus 17/138, respectively, with RR 0.85 (95% CI 0.44 to 1.65). Neurological complications were reported in one participant (in the IVR arm).^12^ Readmissions were not reported in any study (online supplemental table S6a,b).

### Subgroup analyses

Subgroup analysis for Africa and Asia showed no events in the one study from Asia (online supplemental figure S3a,b), so a test of heterogeneity was not carried out, and no pooled estimate was calculated. The prespecified subgroup analysis of in-hospital mortality in the subgroups of children <12 months of age and/or children with kwashiorkor was not reported since the data were not disaggregated in more than one trial.^18 19^

### Additional analyses

There was no evidence of heterogeneity between studies with low risk and some concern (p=0.54; online supplemental figure S4a,b).

## Discussion

Our search strategy only yielded one trial, which strictly fulfilled our criteria for comparing a liberal intravenous rehydration versus a controlled oral strategy. For completeness, we included two RCTs in which the rehydration strategies were either more or less conservative with respect to intravenous rehydration.

The systematic review, involving three trials, found a moderate certainty of evidence indicating the estimated effect of using IVR versus OR in children with SAM with severe dehydration ranges from a 54% relative reduction to a 10% relative increase in the risk of in-hospital death. No event of fluid overload was reported in any of the RCTs. Also of relevance, no events of fluid overload were reported in the two excluded studies involving 169 children receiving IVR.^16 17^ The estimated pooled RR estimate of severe hyponatraemia at 24 hours (per study definitions) in IVR versus OR strategies ranged from a relative reduction of 66%–1%.

Although formal evidence to support or refute the superiority of IVR remains limited, our systematic review underscores the paucity of data generated in clinical trials, with only one incorporating a control strategy reflecting the current recommended rehydration guideline. This review thus reflects the limited internal validity of the results for the primary outcome (although indicating some benefit associated with intravenous rehydration) and highlights the major challenges of conducting high-quality research under extremely challenging conditions, given that severe malnutrition is predominantly encountered in highly volatile humanitarian contexts.

### Limitations

A key limitation of the review is that from the available data, only three trials were eligible for inclusion, including 484 children in total and all of our prespecified secondary endpoints were reported in all the RCTs. No grey literature nor abstracts were searched. Although some study designs of excluded studies were close to an RCT (such as sequential intervention design), we felt it was important to be consistent with our PROSPERO registration and ensure only RCTs were included. Within the three studies included in the systematic review, there was a marked heterogeneity of mortality (ranging from 0% in the Bangladeshi trial,^19^ 9.7% (intervention) and 11.6% (control) in the GASTROSAM trial^12^ and 43% (intervention) and 68% (control) in the smallest trial in Kenyan children^18^ (certainty of evidence rated as moderate due to serious imprecision). Overall, there was insufficient evidence to indicate whether IVR results in either superior or inferior efficacy (in-hospital mortality) to those managed on the conservative OR protocol. The finding, with respect to safety, from the combined data from the RCTs included in the meta-analysis found no evidence of adverse events indicating fluid overload with IVR. Subgroup analyses for kwashiorkor or for children under 1 year were not possible since at least one trial did not report data for these groups, and there was limited reporting on longer-term postdischarge outcomes (>28 days). However, taken together, the data from all the available studies (reporting fluid overload) that received liberal IVR (either slow or fast) indicate that the overall finding of no evidence of fluid overload (including 72 children with kwashiorkor who received intravenous rehydration/volume correction) means that the findings of this meta-analysis are likely to extend to these subgroups.

### Implications for practice

This systematic review provides further evidence that concerns about fluid overload in SAM children receiving IVR are incorrect. When considered alongside earlier physiological studies, showing no evidence of cardiac dysfunction, including a subgroup of children with kwashiorkor,^4 5^ whose Frank-Starling curves during rehydration demonstrated preserved fluid responsiveness,^4 17^ these findings provide compelling evidence to allay the commonly held fears over the use of IVR for children with SAM. The GASTROSAM RCT found in the control OR arm that at admission, 79% of children were unable to take OR, resulting in 92% requiring this via a nasogastric tube. Thus, current recommendations led to additional demands on nurses. Although overall mortality (9%) was substantially lower than predicted from similar cohorts, the authors suggest this was a result of the close clinical monitoring afforded by the trial for ethical reasons. Outside of a clinical trial, this would not be possible in hospitals with limited clinical personnel. Thus, a simplification of the rehydration protocol would potentially be easier to implement. At the very least, both options of rehydration should be made available to children with SAM so either could be used in circumstances where there is a stockout of ORS or intravenous fluids. Relevant to the broader population of children hospitalised with acute diarrhoea with severe dehydration (~10% wt loss), a study showed that approximately 20% temporarily fulfilled anthropometric criteria for SAM (MUAC <11.5 cm) but were ‘reclassified’ as undernourished^20^ following rehydration. Thus, through ‘slippage’, the current recommendations may have wider implications, as potentially 20% of non-SAM children could be inappropriately diagnosed as malnourished and rehydrated.

## Conclusion

The finding in this systematic review that intravenous rehydration in children with SAM and severe dehydration did not result in fluid overload should prompt guidance to be reviewed in light of this.

## Supplementary Material

S1 This content has been supplied by the author(s). It has not been vetted by BMJ Publishing Group Limited (BMJ) and may not have been peer-reviewed. Any opinions or recommendations discussed are solely those of the author(s) and are not endorsed by BMJ. BMJ disclaims all liability and responsibility arising from any reliance placed on the content. Where the content includes any translated material, BMJ does not warrant the accuracy and reliability of the translations (including but not limited to local regulations, clinical guidelines, terminology, drug names and drug dosages) and is not responsible for any error and/ or omissions arising from translation and adaptation or otherwise.

## Figures and Tables

**Table 1:** Characteristics of studies included

Author, year, country	Study design	Participants’ characteristics	Description of intervention	Description of control care	Participants in intervention group	Participants in control group	Mortality before hospital discharge
Akech *et al*^18^, Kenya	Phase II randomised controlled trial	61 children over 6 months of age were enrolled. 40 children had hypovolaemic shock secondary to dehydrating diarrhoea (an additional 21 had hypovolaemic shock secondary to sepsis and are not included in this analysis)	RL resuscitation: initial bolus of 10 mL/kg over 30 min, repeated only twice over 1 hour (ie, up to 30 mL/kg in total). Additional boluses (10 mL/kg over 1 hour) were only permitted for oliguria or hypotension. Maximum bolus volumes given were 40 mL/kg. ReSoMal was given to children with significant diarrhoea.	Initial bolus of 15 mL/kg of HSD/5D over 1 hour. A repeat bolus was given once (15 mL/ kg of HSD/5D over 1 hour) if some improvement in features of shock was noted. If no improvement was seen, they received 10 mL/kg whole blood transfusion over 3 hours. ReSoMal was given to children with significant diarrhoea.	21	19	Overall, 22/40 children died. Intervention: 9/21 (42.8%) Control: 13/19 (68.4%).
Alam *et al*^19^, Bangladesh	Open randomised controlled clinical trial.	Malnourished children aged 6−60 months, with a mixed population of severe acute malnutrition (172) and chronic malnutrition (28).	Isotonic intravenous solution at a rate of 100 mL/kg over 6 hours. Intravenous fluid infusion was stopped after 6 hours, and the ongoing stool loss was replaced with a rice-based ORS and continued until the diarrhoea resolved.	Initially, children received 15 mL/kg of an isotonic intravenous solution over 1 hour. If their respiratory and pulse rate slowed down after 1 hour, the intravenous fluid was continued at 15 mL/ kg for another hour. After 2 hours, intravenous fluids were stopped, and a rice-based ORS was provided at 5−10 mL/kg to provide a total of 70 mL/ kg of ORS.	105 (85 SAM)	103 (87 SAM)	0/85 in intervention arm 0/87 in control arm.
Maitland *et al* ^12^, Kenya, Uganda, Niger and Nigeria	Open-label randomised controlled clinical trial.	Severely malnourished children aged 6 months to 12 years with gastroenteritis and severe dehydration with or without hypovolaemic shock.	Children were randomised between two intervention strategies (then combined for analysis as a liberal strategy). (1) Rapid intravenous rehydration: 100 mL/kg RL over 3−6 hours according to age, including boluses (20 mL/ kg) for those with shock). (2) A slower intravenous rehydration regimen (100 mL/kg RL given over 8 hours and no boluses).	ORS (second randomisation between standard WHO ORS (usually given for non-SAM children) or ReSoMal at a rate of 5 mL/kg every 30 min for the first 2 hour followed by 5−10 mL/kg per hour for the next 4−10 hours on alternate hours, with F-75 milk nutrition formula plus boluses (15 mL/ kg) given for shock. A bolus of RL given over 1 hour and repeated if necessary.	134	138	13/134 (9.7%) in intervention; 16/138 in control (11.6%).

HSD/5D, half-strength Darrow’s/5% dextrose; ORS, oral rehydration solution; ReSoMal, oral rehydration solution for malnutrition; RL, Ringer’s lactate; SAM, severe acute malnutrition.

**Table 2:** Evidence profile for outcomes from meta-analysis

Outcome	Meta-analysis results, risk ratio (95% CI)	Certainty of evidence	Risk of bias	Inconsistency	Indirectness	Imprecision	Publication bias
In-hospital mortality	0.71 (0.46 to 1.10)	Moderate due to serious imprecision.	Results from trials with some concern and low risk are concordant so no rating down.	Visual exploration of the forest plot and heterogeneity statistic I^2^=0% indicated no inconsistency.	The populations and interventions were deemed similar enough that no rating down was needed.	95% CI of overall effect estimate crosses null so rating down one level for imprecision.	A check of clinical trial registries compared with publications included in this review does not suggest publication bias so no rating down was needed.
Heart failure or fluid overload events	0.99 (0.1 to 9.35)	Moderate, due to serious imprecision.	Results from trials with some concern and low risk are concordant so no rating down.	Visual exploration of the forest plot and heterogeneity statistic I^2^=0% indicated no inconsistency.	The populations and interventions were deemed similar enough that no rating down was needed.	95% CI of the overall effect estimate crosses null so rating down one level for imprecision.	
The hyponatraemia at 24 hours	0.66 (0.44 to 0.99)	Moderate, due to serious inconsistency.	Results from trials with some concern and low risk are concordant so no rating down	Visual exploration of the forest plot and heterogeneity statistic I^2^=0% indicated no inconsistency. However, definitions were not consistent between studies so rating down one.	The populations and interventions were deemed similar enough that no rating down was needed.	95% CI of overall effect estimate does not cross null so no rating down was needed.	
Hypokalaemia at 24 hours	1.16 (0.93 to 1.46)	Low, due to serious imprecision and inconsistency.	Results from trials with some concern and low risk are concordant so no rating down.	Visual exploration of the forest plot and heterogeneity statistic I^2^=0% indicated no inconsistency. However, definitions were not consistent between studies so rating down one.	The populations and interventions were deemed similar enough that no rating down was needed.	95% CI of overall effect estimate crosses null so rating down one level for imprecision	

## Data Availability

Data are available upon reasonable request. The data included in this systematic review are available from published work. However, the summary dataset will be shared upon reasonable request.

## What is Already Known on this Topic

⟹ International guidelines advise against giving intravenous rehydration to children with severe acute malnutrition and severe dehydration, unless the child is in shock, due to concerns about fluid overload.
⟹ Evidence to support this recommendation is weak, specifically in children in Africa with severe dehydration due to diarrhoea.

## What this Study Adds

⟹ We systematically reviewed evidence from 484 children in three randomised trials and estimated that intravenous rehydration resulted in a 54% relative reduction to a 10% relative increase in the risk of death.
⟹ No evidence of fluid overload or cardiac failure was reported in any trial.

## How this Study Might Affect Research, Practice or Policy

⟹ The guidance on intravenous rehydration should be reconsidered in light of the findings of this review, considering the safety of intravenous rehydration.
⟹ Simplification of the rehydration guidelines for severe dehydration to remove the distinction between malnourished and non-malnourished children would facilitate ease of implementation.
