# Supplementary material for: Intravenous rehydration in children with severe malnutrition and severe dehydration: a systematic review and meta-analysis
Source: Arch Dis Child. Author manuscript; Available in PMC 2026 Jul 27. (PMC7619270; doi:10.1136/archdischild-2026-330358)
Supplement: S1 — This content has been supplied by the author(s). It has not been vetted by BMJ Publishing Group Limited (BMJ) and may not have been peer-reviewed. Any opinions or recommendations discussed are solely those of the author(s) and are not endorsed by BMJ. BMJ disclaims all liability and responsibility arising from any reliance placed on the content. Where the content includes any translated material, BMJ does not warrant the accuracy and reliability of the translations (including but not limited to local regulations, clinical guidelines, terminology, drug names and drug dosages) and is not responsible for any error and/ or omissions arising from translation and adaptation or otherwise. [file EMS216165-supplement-S1.docx]

## Supplementary material

Contents

[Supplementary material	1](#_Toc214456134)

[Table S1: Search Strategy for Medline	2](#_Toc214456135)

[Table S2: Search Strategy for Embase	3](#_Toc214456136)

[Table S3: Search Strategy for CINAHL	4](#_Toc214456137)

[Table S4: Search Strategy for Cochrane Database	6](#_Toc214456138)

[Table S5: Details of the studies excluded from the analysis	7](#_Toc214456139)

[Table S6a: Characteristics of included studies	9](#_Toc214456140)

[Table S6b: Description of the inventions given in included studies	11](#_Toc214456141)

[Table S7: Availability of the Primary and Secondary outcomes of included studies	15](#_Toc214456142)

[Table S8: Summary of Findings table	17](#_Toc214456143)

[Figure S1: Subgroup analysis of hyponatremia at 24 hours by definition of hyponatremia a) risk ratio, b) risk difference	18](#_Toc214456144)

[Figure S2: Subgroup analysis of hypokalemia at 24 hours by definition of hypokalemia a) risk ratio, b) risk difference	18](#_Toc214456145)

[Figure S3: Subgroup analysis forest plot of in-hospital mortality by geographic region of study a) risk ratio, b) risk difference	19](#_Toc214456146)

[Figure S4: Sensitivity analysis of in-hospital mortality based on Risk of Bias assessment of study a) risk ratio b) risk difference	20](#_Toc214456147)

### Table S1: Search Strategy for Medline

| **#** | **Query** |
| --- | --- |
| 1 | (Fluid therapy or fluid-therapy).tw. |
| 2 | ((intraven* or IV or parenteral) adj infusion).tw. |
| 3 | ((intraven* or IV or parenteral) adj administration).tw. |
| 4 | exp administration, intravenous/ or infusions, intravenous/ |
| 5 | Fluid Therapy/ |
| 6 | (infant$ or baby or babies or child$ or boy$ or girl$ or preschool$ or pre-school$).tw. |
| 7 | exp child/ or infant/ |
| 8 | (malnutrition or mal-nutrition or malnourish$ or wasted or wasting or emaciat$ or undernutrition or undernourish$ or marasmus$ or kwashiorkor).tw. |
| 9 | exp Severe Acute Malnutrition/ |
| 10 | Protein-Energy Malnutrition/ |
| 11 | (dehydration or hypovolemia).tw. |
| 12 | exp Dehydration/ |
| 13 | (diarrh$ or gastroenteritis or gastrointestinal infection$ or enteritis).tw. |
| 14 | exp Diarrhea/ |
| 15 | 1 or 2 or 3 or 4 or 5 |
| 16 | 6 or 7 |
| 17 | 8 or 9 or 10 |
| 18 | 11 or 12 |
| 19 | 13 or 14 |
| 20 | 15 and 16 and 17 and 18 and 19 |

### Table S2: Search Strategy for Embase

| **#** | **Query** |
| --- | --- |
| 1 | (Fluid therapy or fluid-therapy).tw. |
| 2 | ((intraven* or IV or parenteral) adj infusion).tw. |
| 3 | ((intraven* or IV or parenteral) adj administration).tw. |
| 4 | exp intravenous drug administration/ |
| 5 | exp fluid therapy/ |
| 6 | (infant$ or baby or babies or child$ or boy$ or girl$ or preschool$ or pre-school$).tw. |
| 7 | exp child/ or exp infant/ |
| 8 | (malnutrition or mal-nutrition or malnourish$ or wasted or wasting or emaciat$ or undernutrition or undernourish$ or marasmus$ or kwashiorkor).tw. |
| 9 | exp malnutrition/ |
| 10 | exp protein calorie malnutrition/ |
| 11 | exp wasting syndrome/ or exp kwashiorkor/ or exp marasmus/ |
| 12 | (dehydration or hypovolemia).tw. |
| 13 | exp dehydration/ |
| 14 | (diarrh$ or gastroenteritis or gastrointestinal infection$ or enteritis).tw. |
| 15 | exp diarrhea/ |
| 16 | 1 or 2 or 3 or 4 or 5 |
| 17 | 6 or 7 |
| 18 | 8 or 9 or 10 or 11 |
| 19 | 12 or 13 |
| 20 | 14 or 15 |
| 21 | 16 and 17 and 18 and 19 and 20 |

### Table S3: Search Strategy for CINAHL

| S19 | S14 AND S15 AND S16 AND S17 AND S18 | Expanders - Apply equivalent subjects  Search modes - Proximity |
| --- | --- | --- |
| S18 | S12 OR S13 | Expanders - Apply equivalent subjects  Search modes - Proximity |
| S17 | S10 OR S11 | Expanders - Apply equivalent subjects  Search modes - Proximity |
| S16 | S7 OR S8 OR S9 | Expanders - Apply equivalent subjects  Search modes - Proximity |
| S15 | S5 OR S6 | Expanders - Apply equivalent subjects  Search modes - Proximity |
| S14 | S1 OR S2 OR S3 OR S4 | Expanders - Apply equivalent subjects  Search modes - Proximity |
| S13 | (MH "Diarrhea") | Expanders - Apply equivalent subjects  Search modes - Proximity |
| S12 | diarrh* or gastroenteritis or gastrointestinal infection* or enteritis | Expanders - Apply equivalent subjects  Search modes - Proximity |
| S11 | (MH "Dehydration") | Expanders - Apply equivalent subjects  Search modes - Proximity |
| S10 | dehydration or hypovolemia | Expanders - Apply equivalent subjects  Search modes - Proximity |
| S9 | (MH "Wasting Syndrome+") | Expanders - Apply equivalent subjects  Search modes - Proximity |
| S8 | (MH "Malnutrition+") OR (MH "Protein-Energy Malnutrition+") OR (MH "Kwashiorkor") | Expanders - Apply equivalent subjects  Search modes - Proximity |
| S7 | malnutrition or mal-nutrition or malnourish* or wasted or wasting or emaciate* or undernutrition or undernourish* or marasmus* or kwashiorkor | Expanders - Apply equivalent subjects  Search modes - Proximity |
| S6 | (MH "Infant+") OR (MH "Child+") | Expanders - Apply equivalent subjects  Search modes - Proximity |
| S5 | infant* or baby or babies or child* or boy* or girl* or preschool* or pre-school* | Expanders - Apply equivalent subjects  Search modes - Proximity |
| S4 | (MH "Administration, Intravenous+") OR (MH "Intravenous Therapy+") OR (MH "Infusions, Intravenous") | Expanders - Apply equivalent subjects  Search modes - Proximity |
| S3 | (intraven* or IV or parenteral) W1 administration | Expanders - Apply equivalent subjects  Search modes - Proximity |
| S2 | (intraven* or IV or parenteral) W1 infusion | Expanders - Apply equivalent subjects  Search modes - Proximity |
| S1 | “Fluid therapy” or fluid-therapy | Expanders - Apply equivalent subjects  Search modes - Proximity |

### Table S4: Search Strategy for Cochrane Database

|  | **Search:** |
| --- | --- |
| #1 | ("Fluid therapy" or "fluid-therapy" or intraven* NEAR infusion or IV NEAR infusion or parenteral NEAR infusion or intraven* NEAR administration or IV NEAR administration or parenteral NEAR administration):ti,ab,kw (Word variations have been searched) |
| #2 | MeSH descriptor: [Administration, Intravenous] this term only |
| #3 | (infant* or baby or babies or child* or boy* or girl* or preschool* or pre-school*):ti,ab,kw (Word variations have been searched) |
| #4 | MeSH descriptor: [Child] explode all trees |
| #5 | MeSH descriptor: [Infant] explode all trees |
| #6 | MeSH descriptor: [Severe Acute Malnutrition] explode all trees |
| #7 | MeSH descriptor: [Dehydration] explode all trees |
| #8 | (diarrh$ or gastroenteritis or gastrointestinal infection$ or enteritis):ti,ab,kw (Word variations have been searched) |
| #9 | (malnutrition or mal-nutrition or malnourish* or mal‐nourish* or wasted or wasting or emaciat* or undernutrition or under‐nutrition or undernourish* or under‐nourish* or marasmus* or kwashiorkor):ti,ab,kw (Word variations have been searched) |
| #10 | (dehydration or hypovolemia):ti,ab,kw (Word variations have been searched) |
| #11 | MeSH descriptor: [Diarrhea] explode all trees |
| #12 | #1 OR #2 |
| #13 | #3 OR #4 OR #5 |
| #14 | #6 OR #9 |
| #15 | #7 OR #10 |
| #16 | #8 OR #11 |
| #17 | #12 AND #13 AND #14 AND #15 AND #16 |

### Table S5: Details of the studies excluded from the analysis

| **Author, year, country** | **Study design** | **Participants characteristics** | **Description of intervention** | **Description of control care** | **Participants in intervention group** | **Participants in control group** | **Mortality before hospital discharge** |
| --- | --- | --- | --- | --- | --- | --- | --- |
| Bangladesh | Before-and-after observational study | Children aged 0–5 years  severely malnourished with  diarrhoea with various degrees of dehydration | Patients with some dehydration: oral rehydration according to WHO guidelines Patients with severe dehydration: patients were rehydrated with 100 mL/kg of isotonic intravenous fluid infused over 6 h in infants under 12 months and over 3h in older children | Patients with some dehydration: rice-based ORS 10 mL per kg per h for the first 2 h, then 5 mL per kg per h for the next 10 h.  Patients with severe dehydration: initial rehydration by isotonic IV fluid 20 mL/kg for 1 h then 10 mL/kg for 1 h. ORS 10 mL/kg/ h was started after 1 h. | 293 | 334 | 49/293 in intervention group; 30/334 in the control group |
| Alam, 2009 Bangladesh | The study was conducted in two distinct phases:  1. Phase I: safety evaluation of rapid intravenous rehydration in severely dehydrated and malnourished children through an observational study design without a control group to assess the safety of administering rapid intravenous rehydration  2.Phase II: comparative efficacy of three ORS formulations, following the initial safety assessment, through an RCT | Children with severe malnutrition (one third of patients had acute malnutrition),  aged 6 to 60 months, with  acute watery diarrhoea of less than  48 hours and  stool dark-field microscopy demonstrating presence of Vibrio cholerae.  149/175 patients had severe dehydration | Children with severe dehydration (149/175) were initially rehydrated with an IV ‘‘cholera saline’’ solution containing Na+ 133, K+ 13, Cl- 98, and acetate 48 (all in mmol/L) 100 mL/kg, for 4 to 6 hours.  After IV rehydration, all patients (175) were randomised to 3 formulations of ORS with the same salts composition but with different substrate composition: 1) glucose-ORS, 2) glucose-ORS plus 50 g/L of amylase-resistant starch, or 3) rice-ORS | Not applicable | 149 severely dehydrated children underwent phase 1.  175 children were then randomised to the three ORS formulations:  1) 58 to glucose-ORS arm,  2) 59 to glucose-ORS plus  50 g/L of amylase-resistant starch, and  3) 58 to rice-ORS | Not applicable | 0/175 |
| Obonyo, 2017  Kenya and Uganda | Prospective observational study | Severely malnourished  Children,  aged 6-60 months, with hypovolemic shock secondary to acute  gastroenteritis | 10 ml/kg/h of RL over maximum 5 h. They received rehydration volume replacement without an initial fluid bolus. Children were switched to oral rehydration once they were able to tolerate oral intake or nasogastric fluids (4/9). | Bolus of 15 ml/kg of RL over 1 h, with the option of repeating it once (15 ml/kg) if signs of shock persist, followed by HSD/5D at a rate of 4 ml/kg/h. Children were switched to oral rehydration once they were able to tolerate oral intake (1/11). | 9 | 11 | 3/9 in intervention group; 5/11 in control  group  Day 28 mortality 5/9 vs 9/11 |

### Table S6a: Characteristics of included studies

| **Author (Year, journal)** | **Study design** | **Country** | **SAM definition** | **Dehydration definition** | **Inclusion criteria** | **Exclusion criteria** | **Kwashiorkor** |
| --- | --- | --- | --- | --- | --- | --- | --- |
| Akech, 2010 | Phase II randomized controlled trial | Kenya | Weight for height Z (WHZ) score <-3 or weight for height  percentile 70% or  Mid- Upper Arm Circumference (MUAC) <11.0cm or oedema involving at least both feet | Dehydrating diarrhea defined as: ≥6 watery stools per day Shock definition: WHO shock criteria were amended to include children with one or more of the following: CRT > 2 seconds, lower limb temperature gradient, weak pulse volume, prolonged capillary refill > 2 seconds, deep ‘acidotic’ or ‘Kussmauls’ breathing, creatinine >80 μmol/L, or depressed conscious state if present after correction of hypoglycemia | Children aged over 6 months with severe malnutrition with evidence of shock | 1) Severe anemia with hemoglobin less or equals to 5g/dl 2) Pulmonary oedema (defined as clinical evidence of presence of fine crepitations in both lung fields plus oxygen saturation <90% in  air) 3) Raised intra-cranial pressure or known congenital heart disease | Yes, 12 patients in total |
| Alam, 2020 | Open randomized controlled clinical trial | Bangladesh | Children with weight for age (WAZ) or weight for length/height Z (WHZ) scores of less than −3, or with bipedal oedema | Severe dehydration according to a modified version of WHO guidelines | Children aged 6-60 months, severely underweight or with severe acute malnutrition, acute diarrhea (>= 3 episodes in less than 24 hours), and  severe dehydration | Dysentery, severe and very severe pneumonia, suspected severe sepsis or septic shock, suspected meningitis, antibiotic use for the current illness before admission. | Yes, 49 patients in total |
| Maitland, 2025 | Open label randomized controlled clinical trial | Kenya, Uganda, Niger, Nigeria | Children with one or more of the following:  MUAC <11.5cm; WHZ <-3; Kwashiorkor | Signs of dehydration as per WHO definition: two or more of decreased conscious level (AVPU<A); sunken eyes; reduced skin turgor (abdominal skin pinch goes back slowly (>2 seconds)); unable to take or retain oral fluids | Children aged 6 months to 12 years with SAM and Gastroenteritis and severe dehydration | Diarrhea lasting more than 14 days; known congenital or rheumatic heart disease; refusal of consent | Yes, 11 patients in total |

### Table S6b: Description of the inventions given in included studies

| **Author (Year, journal)** | **Participants age** | **Participants characteristics** | **Description of intervention** | **Description of control care** | **Number of participants in intervention group (total number and number <12 months of age)** | **Number of participants in control group (total number and number <12 months of age** | **Outcomes** |
| --- | --- | --- | --- | --- | --- | --- | --- |
| Akech, 2010 | Children over 6 months | 61 were enrolled. Forty children had hypovolemic shock secondary to dehydrating diarrhea (included in the analysis) and 21 had hypovolemic shock, presumed to have been secondary to sepsis or a sepsis-like syndrome (not included in the analysis). | Ringers Lactate (RL) or albumin (HAS) resuscitation: initial bolus of 10 ml/kg over 30 minutes, repeated only twice over one hour (i.e. up to 30 ml/kg in total) if clinical reassessment demonstrated any of the features. Additional boluses (10 ml/kg over one hour) were only permitted if oliguria or hypotension. Maximum bolus volumes given were 40 ml/kg.  Rehydration SOlution for Malnutrition (ReSoMal) was given to children with significant diarrhea (>6 loose stools/day) | WHO fluid resuscitation regime: initial bolus of 15 ml/kg of half-strength Darrow’s in 5% dextrose (HSD/5D) over one hour. Repeat bolus was given once (15 ml/kg of HSD/5D over 1 hour) if some improvement in features of shock noted. If no improvement was seen, they received 10 ml/kg whole blood transfusion over 3 hours. HSD/5D was an option in case of severe dehydration & shock, and in case of presumptive septic shock with no dehydration. ReSoMal (Rehydration SOlution for Malnutrition) was given to children with significant diarrhea (>6 loose stools/day) | 21 patients received RL (no data on <12 months) | 19 (no data on <12 months) | Primary outcome measurement was the resolution of features of shock at 8 and 24 hours. Secondary outcomes included incidence of  adverse events and mortality |
| Alam, 2020 | 6-60 months | Malnourished children aged 6-60 months, with a mixed population of severe acute malnutrition (172) and chronic malnutrition (28) | IV Cholera saline (sodium 133 mmol/L, chloride 98 mmol/L, potassium 13 mmol/L and bicarbonate 48 mmol/L) at a rate of 100 mL/kg over six hours. Ongoing stool losses were replaced with a rice-based oral rehydration solution that was prepared locally (5-10 mL/kg was fed orally or through a nasogastric tube after each watery stool). Intravenous fluid infusion was stopped after six hours, and the ongoing stool loss was replaced with the rice-based ORS and continued until the diarrhea resolved. | Initially, they received 15 mL/kg of the intravenous cholera saline detailed before over one hour and monitored every 10-15 minutes. If their respiratory and pulse rate slowed down after one hour, the intravenous fluid was continued at 15 ml/kg for another hour. After two hours, the intravenous fluid was stopped, and the intravenous line was left open. The rehydration process still had an estimated deficit of 70 ml/kg at this point, and the rice-based ORS was started through a nasogastric tube at 5-10 mL/kg. | 105 (85 SAM) (no data on <12 months) | 103 (87 SAM) (no data on <12 months) | Administration of unscheduled IV rehydration, Treatment failure defined as not achieving rehydration after 6 hours in rapid group, after 12 hours in slow group, fluid overload, heart failure |
| Maitland, 2025 | 6 months to 12 years | Severely malnourished children aged 6 months to 12 years with gastroenteritis and severe dehydration with or without hypovolemic shock | Children were randomized between two intervention strategies (then combined for analysis as a liberal strategy). 1) rapid IV rehydration as per WHO Plan C (usually for non-SAM children) (100 ml/kg Ringer’s Lactate (RL) over 3-6 hours according to age including boluses (20 ml/kg) for those with shock) 2) A slower IV rehydration regimen (100 ml/kg RL given over 8 hours and no boluses) | WHO standard of care for children with SAM and severe dehydration: ORS (second randomization between standard WHO ORS (usually given for non-SAM children) or WHO SAM-recommended low-sodium ReSoMal, at a rate of 5 ml/kg every 30 min for the first 2 h followed by 5–10 ml/kg per h for the next 4–10 h on alternate hours, with F-75 milk nutrition formula plus boluses (15ml/kg) given for shock. Bolus of Ringer's lactate given over 1 hour and repeated if necessary. | 134 | 138 | Mortality at 96hr; mortality at 28 days; fluid overload events (pulmonary edema or heart failure) during admission; change in plasma sodium levels; electrolyte abnormalities at 8 hours; weight and MUAC change to day 3 and day 7. |

### Table S7: Availability of the Primary and Secondary outcomes of included studies

| **Author (Year, journal)** | **Mortality before hospital discharge** | **Development of pulmonary oedema or heart failure ( fluid overload)** | **Development of shock requiring intravenous fluids** | **Development of neurological complications (convulsions or decrease in conscious level)** | **Perturbations of electrolyte abnormalities (severe hyponatremia** | **Severe hypernatremia > 140 mmol/L or hypokalemia** | **Day-28 mortality** | **Readmission to hospital** | **Were there any subgroup analyses performed** |
| --- | --- | --- | --- | --- | --- | --- | --- | --- | --- |
| Akech, 2010 | Intervention: 9/21  Control:13/19 | Intervention group: RL: 0/21 Control group: HSD/5D: 0/19 | Not available (NA) for the subset of children with diarrhea | NA | Only baseline data was provided and then mean sodium concentrations. There were no differences in the mean sodium concentration at admission versus 8 hours, and 24 hours between intervention and control groups | Only baseline data was provided, ie electrolyte abnormalities at recruitment but no follow up data after interventions were administered | NA | NA | No |
| Alam, 2020 | 0/105 in intervention arm  0/103 in control arm | 0/105 in intervention arm  0/103 in control arm | NA | NA | 24 hours: 10/80 (12.5%) in intervention; 12/82(14.6%) in control. | Hypokalemia at 24 hours: 48/80(60%) intervention; 45/82 (54.9%) control. | NA | NA | Yes (for SAM and chronic malnutrition subgroups) |
| Maitland, 2025 | 13/134 in intervention; 16/138 in control | 0/134 in intervention arm; 0/138 in control arm | 6/134 in the intervention arm; 11/138 in the control arm | 10/134 in intervention arm; 18/138 in the control arm | 8hours: 20/128 (16%) in intervention; 58/126 (45%) in control.  24 hours: 21/127 (17%) in intervention; 35/126 (27%) in control. | Hypernatremia (only reported >145mmols/L). 8 hours: 2/128 (2%) intervention; 2/126 (2%) control. 24 hours: 6/127 (5%) intervention; 3/126 (2%) control. Hypokalemia. 8 hours 57/128 (45%) intervention; 40/126 (31%) control. 24 hours 36/127 (29%) intervention; 26/126 (20%) control. | 14/134 (10%) in intervention group; 17/138 (12%) in control. | NA | Yes (for age<1 years; conscious level (AVPU); ORS randomization; respiratory distress). |

### Table S8: Summary of Findings table

|  | Estimated risks | |  |  |  |  |
| --- | --- | --- | --- | --- | --- | --- |
| Outcomes | Control risks | Intervention risks | Relative effect (95% CI) | Absolute effect (95%CI) | Number of participants (studies) | Quality of the evidence |
| In hospital mortality | 29/244 (11.9%) | 22/240 (9.2%) | 0.71 (0.46, 1.10) | -3.2% (-10.4, 3.9) | 488 (3) | Moderate |
| Heart failure or fluid overload events | 0/244 (0%) | 0/240 (0%) | 0.99 (0.10, 9.35) | Not estimated | 488 (3) | Moderate |
| Hyponatraemia at 24 hours | 27/208 | 31/207 | 0.66 (0.44, 0.99) | -6.8% (-15.7, 2.1) | 415 (2) | Moderate |
| Hypokalemia at 24 hours | 71/207 | 84/207 | 1.16 (0.93, 1.46) | 6.9% (-1.8, 15.5) | 415 (2) | Low |

### Figure S1: Subgroup analysis of hyponatremia at 24 hours by definition of hyponatremia a) risk ratio, b) risk difference

| a)   | b)  |
| --- | --- |

### Figure S2: Subgroup analysis of hypokalemia at 24 hours by definition of hypokalemia a) risk ratio, b) risk difference

| a)   | b)   |
| --- | --- |

### Figure S3: Subgroup analysis forest plot of in-hospital mortality by geographic region of study a) risk ratio, b) risk difference

| a)   | b) |
| --- | --- |

### Figure S4: Sensitivity analysis of in-hospital mortality based on Risk of Bias assessment of study a) risk ratio b) risk difference

| a)    | b) |
| --- | --- |
